# Burden, patterns, and challenges of HIV-associated malignancies in Nigeria: a narrative review

**DOI:** 10.3389/fonc.2026.1959750

**Published:** 2026-10-16

**Authors:** Olugbenga Silas, Chuwang Nyam, Yop Tsok, Michael Eshioramine, Noel Nnaegbuna, William Golit, Adeyemi Okunowo, Chidubem Osuagwu, Juliet Offor, Izu Achusi, Tolu Beneye, Maradona Isikhuemen, Victor Ekanem, Lawson Ekpe, Patience Odusolu, Andrea Akinjo, TAGCReC, Fatima Rasheed, Folusakin Ayoade, Bala Audu, Ahmed Saad, Ayodele Omotoso, Sophia George

**Affiliations:** 1 University of Jos, Jos, Plateau State, Nigeria; 2 Plateau State Specialist Hospital, Jos, Plateau State, Nigeria; 3 Department of Obstetrics and Gynecology, College of Medicine, University of Lagos, Lagos, Nigeria; 4 Department of Anatomic and Molecular Pathology, College of Medicine, University of Lagos, Lagos, Nigeria; 5 National Obstetric Fistula Centre, Abakaliki, Ebonyi State, Nigeria; 6 Department of Obstetrics and Gynecology, Federal Medical Center, Abuja, Nigeria; 7 Department of Pathology, Federal Medical Center, Abuja, Nigeria; 8 University of Benin Teaching Hospital, Benin, Edo State, Nigeria; 9 Department of Chemical Pathology, University of Calabar, Calabar, Nigeria; 10 Department of Obstetrics and Gynecology, University of Calabar, Calabar, Nigeria; 11 Sylvester Comprehensive Cancer Center, University of Miami Miller School of Medicine, Miami, FL, United States; 12 Federal University of Health and Science, Azare, Bauchi State, Nigeria

**Keywords:** AIDS-defining cancers, cancer surveillance, HIV-associated malignancies, Nigeria, non-AIDS-defining cancers

## Abstract

**Background:**

Nigeria bears one of the world’s largest burdens of HIV, with ongoing expansion of antiretroviral therapy (ART) significantly improving the survival of people living with HIV (PLHIV). This epidemiologic shift has changed the pattern of morbidity and mortality toward chronic conditions, especially HIV-associated malignancies (HIVAMs), including both AIDS-defining malignancies (ADMs) and non-AIDS-defining malignancies (NADMs). Despite their increasing significance, the national burden, patterns, and systemic challenges related to HIVAMs in Nigeria remain poorly understood and characterized.

**Methods:**

We conducted a narrative review of peer-reviewed literature, cancer registry reports, and grey literature published between January 2000 and December 2024. Searches were carried out in PubMed/MEDLINE, Embase, African Journals Online, and Google Scholar, complemented by national reports from the National Agency for the Control of AIDS, the Federal Ministry of Health, and the National System of Cancer Registries. Eligible sources reported cancer occurrence among PLHIV or HIV prevalence among cancer patients in Nigeria. Findings were synthesized narratively due to significant heterogeneity across study designs and outcomes.

**Results:**

Seventy-three sources met the inclusion criteria, and these were primarily hospital-based and retrospective studies. ADMs remain the most common cancers among PLHIV, notably invasive cervical cancer, Kaposi sarcoma, and non-Hodgkin lymphoma, with notable regional differences. Expansion of ART has been linked to a decline in Kaposi sarcoma incidence, while the burden of cervical cancer remains high, driven by persistent oncogenic HPV infection and limited screening coverage among women living with HIV (WLWH). Emerging NADMs, such as hepatocellular carcinoma, Hodgkin lymphoma, breast cancer, and ocular surface squamous neoplasia, reflect improved survival, aging, chronic immune dysregulation, and co-infections like Hepatitis B and C. Across these cancers, late-stage presentation and poor outcomes are common, largely due to fragmented HIV–cancer surveillance, inadequate pathology and radiotherapy capacity, limited molecular diagnostics, high out-of-pocket treatment costs, and weak integration of HIV and oncology services.

**Conclusion:**

HIV-associated cancers pose a major yet often overlooked public health issue in Nigeria. Enhancing integrated HIV–cancer surveillance, increasing prevention and early detection efforts through ART programs, upgrading diagnostic and treatment facilities, and utilizing digital and molecular innovations are essential for decreasing preventable cancer-related morbidity and mortality among PLHIV in Nigeria.

## 1 Introduction

The global population of people living with HIV (PLHIV) increased from 27.2 million in 2000 to 40.8 million in 2024, with Nigeria contributing disproportionately to this number, with about 4.5 -5.0% of the cases, and ranking fourth worldwide in the number of PLHIV (1, 2). Although Nigeria has enjoyed a 2.3% annual population growth over the past decade, Nigeria continues to carry a substantial HIV burden, with current estimates of approximately 1.9–2.0 million PLHIV (2–4). Earlier figures of about 3.2 million were revised downward following the more rigorous 2018 Nigeria HIV/AIDS Indicator and Impact Survey (NAIIS), which reduced national prevalence from 2.8% to 1.4%; the ~1.9–2.0 million estimate has since remained relatively stable across recent UNAIDS/Spectrum and modeling data. This reflects improved measurement rather than a true epidemiological decline, even as sustained HIV-prevention campaigns and ART expansion have markedly improved survival and allowed more individuals to live longer, healthier lives (3). As PLHIV continue to live longer, the epidemiologic profile has shifted from opportunistic infections to non-communicable diseases, including cancers (5). These comorbidities are largely driven by chronic inflammation, ongoing immunodeficiency, viral co-infections, and behavioral risks (6). Consequently, Nigeria now faces a dual burden of HIV and cancer, collectively referred to as HIV-associated malignancies (HIVAM).

The common HIVAMs associated with progression to AIDS in surveillance systems among these individuals are the traditional AIDS-defining malignancies (ADMs), namely Kaposi sarcoma (KS), invasive cervical cancer (ICC), and non-Hodgkin lymphoma (NHL) (7–9). Furthermore, PLHIV face an increased risk of several non-AIDS-defining malignancies (NADMs), such as Hodgkin lymphoma, anal cancer, liver cancer, lung cancer, oral cancer, and breast cancer, compared to HIV-negative populations (7–9). Chronic immune activation, persistent inflammation, co-infections, and prolonged survival partly contribute to the elevated risk of NADMs. These cancers exhibit complex risk patterns influenced by aging, tobacco and alcohol use, co-infections, and ongoing inflammatory processes (6–10). In Nigeria, this increase in cancer burden can be accounted for by co-infections with human papillomavirus (HPV), hepatitis B endemicity, and elevated HHV-8 seroprevalence interact in a syndemic fashion with ongoing immunosuppression, late ART initiation, poor adherence, and delayed HIV diagnosis (11, 12).

Despite the rising burden of HIVAMs in Nigeria, accurately measuring the national scope and distribution remains challenging. Nigeria has begun developing a national cancer surveillance system through the National System of Cancer Registries (NSCR) and the National Institute for Cancer Research and Treatment (NICRAT), but limitations in coverage, data quality, reporting, and coordination hinder comprehensive monitoring (13, 14). Reports from the Nigerian National Agency for the Control of AIDS (NACA) and the Federal Ministry of Health (FMOH) consistently reveal systemic gaps, such as poor integration between HIV and cancer data, shortages of trained oncology and pathology staff, and significant disparities in access to essential diagnostic and treatment services like radiotherapy, molecular diagnostics, and immunohistochemistry (3, 13, 14). As a result,existing data on HIVAMs comes from single-center or regional hospital studies, which are useful but insufficient for reliable national estimates. An integrated, strong HIV–cancer surveillance system is urgently needed to fill these gaps.

This narrative review addresses this critical gap by systematically synthesizing available information on the burden, epidemiological patterns, and systems-related challenges of HIVAMs in Nigeria. By consolidating findings from peer-reviewed literature, cancer registries, and policy documents from 2000 to 2024, we aim to provide a comprehensive assessment of the current state of knowledge and identify priority areas for research, policy, and programmatic action to improve outcomes for Nigerians affected by HIV and cancer.

## 2 Methods

### 2.1 Search strategy

We conducted a comprehensive literature search in accordance with established guidance for narrative reviews (15). Searches were performed in PubMed/MEDLINE, Embase, African Journals Online (AJOL), and Google Scholar databases. Grey literature sources and registry data were also systematically reviewed (3, 13, 14). The electronic search strategy combined Medical Subject Headings (MeSH) and free-text terms: (“HIV” OR “human immunodeficiency virus” OR “AIDS” OR “acquired immunodeficiency syndrome”) AND (“cancer” OR “malignancy” OR “neoplasm” OR “tumor” OR “Kaposi sarcoma” OR “lymphoma” OR “cervical cancer”) AND (“Nigeria” OR “Nigerian”). Reference lists of included articles were screened to identify additional relevant studies.

### 2.2 Grey literature and national data sources

To improve completeness and national representativeness, we systematically reviewed grey literature and registry reports, including:

1. National-level HIV and cancer documents: NACA reports and technical briefs, including the Nigeria HIV/AIDS Indicator and Impact Survey (NAIIS) and HIV epidemiology fact sheets (3, 14, 16), and FMOH national HIV and cancer control strategy documents and reports on ART program performance and cancer control planning (14).

ii. Population-based and hospital-based cancer registries: Ibadan Population-Based Cancer Registry (IBCR), one of Africa’s oldest registries (17, 18), Hospital-based cancer registries at Jos University Teaching Hospital (JUTH), Lagos University Teaching Hospital (LUTH), Ahmadu Bello University Teaching Hospital (ABUTH), University of Nigeria Teaching Hospital (UNTH), Aminu Kano Teaching Hospital (AKTH), and other regional registries where available (19–22). This cancer registry list constitutes a reliable representation of institution-managed cancer epidemiological data at tertiary institutions in Nigeria.

From these sources, we extracted data on cancer incidence and site distribution, where reported, HIV prevalence among cancer patients, patterns of HIVAMs over time, regional differences, and health system capacity. While HIV status is not yet routinely captured in most cancer registries, qualitative and quantitative data on likely HIV-associated malignancies (e.g., KS, ICC, lymphoma) were also extracted where registry reports or institutional summaries made this explicit.

### 2.3 Eligibility criteria

Inclusion criteria

1. Original research (cross-sectional, case–control, cohort studies), case series (≥5 cases), systematic reviews, and meta-analyses.
2. Studies reporting cancer occurrence among PLHIV or HIV prevalence among cancer patients in Nigeria.
3. Relevant grey literature and registry reports, provided they contained original data, summary statistics, or official estimates relevant to HIVAM burden or health system capacity.
4. Publications in English.
5. Publication date between January 2000 and December 2024.

Exclusion criteria

1. Case reports (<5 cases).
2. Studies conducted exclusively outside Nigeria.
3. Papers without original data (editorials, commentaries, non-systematic narrative reviews).
4. Studies addressing only non-malignant HIV-related complications.

### 2.4 Data extraction and synthesis

Two reviewers independently screened titles and abstracts; full texts of potentially eligible studies were retrieved. Discrepancies were resolved through discussion, with adjudication by a third reviewer where consensus was not reached; as this was a narrative synthesis, a formal inter-rater agreement statistic (e.g., Cohen’s κ) was not computed. Extracted variables included study design, setting, sample size, population characteristics, cancer types, HIV prevalence or incidence, clinical stage at diagnosis, treatment patterns, outcomes, and reported barriers to care. From registries and grey sources, we extracted: overall and sex-specific cancer incidence; proportional distribution of major cancer types; qualitative or quantitative information on suspected HIV-associated malignancies; and commentary on diagnostic, treatment, and surveillance capacity.

Given substantial heterogeneity in study designs, populations, and outcomes, we did not perform a meta-analysis. Instead, we used a narrative synthesis approach, organizing findings by malignancy type (ADMs and NADMs), special populations, and key challenge domains (surveillance, diagnosis, treatment, health system integration, and patient-level factors). Summary of key included studies on HIV-associated malignancies is included in **Table 1**.

**Table 1:** Summary of key included studies on HIV-associated malignancies in Nigeria.

Study [ref], design	Setting/period	N	Focus	Key finding	RoB
Kaposi sarcoma					
Akanbi et al. (43) Retrospective cohort	JUTH, Jos (N-central); 2006-2016	14,479 PLHIV (68,168 PY; 67% F)	Kaposi sarcoma	KS incidence fell 2.53 -> 1.58/1,000 PY across expanding ART eligibility.	Low
Akanbi et al. (42) Retrospective cohort	Nigeria (Jos/APIN); 2006-2017	10,788 (65.7% F)	Kaposi sarcoma	KS prevalence 0.59% at entry to HIV care; incidence not yet declined despite program expansion.	Low
Agaba et al. (45) Retrospective	JUTH, Jos; pre-/post-ART	NR - verify	AIDS-related KS	Poor pre-ART outcomes; marked survival gain after ART scale-up.	Mod
Akinde et al. (46) Retrospective clinicopathological	LUTH, Lagos (SW); 14-yr	NR - verify	Kaposi sarcoma	KS a leading ADM; male predominance, young age at presentation.	Mod
Mandong et al. (44) Histopathology review	JUTH, Jos; 16-yr	NR - verify	Kaposi sarcoma	Histopathological patterns of KS in Jos over 16 years.	Mod
Forae & Obaseki (47) Retrospective histopathology	UBTH, Benin City	NR - verify	Kaposi sarcoma	Trends and histopathological patterns of KS in Benin City.	Mod
Asuquo et al. (48) Retrospective	Calabar (S-east)	NR - verify	Kaposi sarcoma	Changing trend of KS in Calabar.	Mod
Kagu et al. (49) Retrospective	Maiduguri (N-east)	NR - verify	AIDS-associated KS	Pattern of AIDS-associated KS in north-eastern Nigeria.	Mod
Ahmed et al. (53) Case series	ABUTH, Zaria (N-west)	<20 - verify	Pediatric AIDS-KS	Distinct clinical presentation of pediatric KS.	High
Silas et al. (50) Registry analysis	JUTH registry, Jos; 1996-2018	Registry - verify	Top-10 cancers	KS among leading cancers; shifting registry patterns over time.	Low
Invasive cervical cancer & HPV					
Musa et al. (26) Multi-institutional cohort	JUTH & LUTH; 2018-2022	239 ICC (47 HIV+, 192 HIV-)	HIV-associated cervical cancer	HIV+ diagnosed ~11 yr earlier; 12-mo OS 67.6% vs 84.1%; lower treatment completion.	Low
Daniel et al. (27) Cross-sectional	Jos (N-central)	NR - verify	Cervical precancer	Prevalence & predictors of precancerous lesions in HIV+ women.	Mod
Anorlu et al. (28) Cross-sectional	LUTH, Lagos (SW)	NR - verify	Cervical cytology	Prevalence of abnormal cervical smears among patients with HIV.	Mod
Awolude & Oyerinde (29) Cross-sectional	Ibadan (SW)	NR - verify	Invasive cervical cancer	Socio-sexual profile, stage at presentation, histology & HIV status.	Mod
Nyam et al. (30) Molecular epidemiology	Jos & Lagos (2 regions)	NR - verify	HPV genotypes	Greater HR-HPV genotype diversity in WLWH with cervical cancer; HPV35 prominent.	Low
Ezechi et al. (58) Cross-sectional	Lagos (NIMR)	NR - verify	Oncogenic HPV	Burden, distribution & risk factors for oncogenic cervical HPV in HIV+ women.	Mod
Akarolo-Anthony et al. (60) Cross-sectional	Abuja	NR - verify	High-risk HPV	HIV-associated high-risk HPV infection among Nigerian women.	Mod
Musa et al. (61) Cross-sectional	Jos	NR - verify	HR-HPV/cytology	HR-HPV & cervical abnormalities in HIV+ women with normal cytology.	Mod
Abdus-Salam et al. (62) Retrospective	Ibadan (UCH)	NR - verify	ICC on radiotherapy	HIV prevalence among cervical cancer patients receiving radiotherapy (revisit).	Mod
Kabuga et al. (59) Systematic review & meta-analysis	Nigeria	Pooled - verify	HPV prevalence/types	Prevalence & type distribution of cervical HPV in Nigerian women.	Low
Atibioke et al. (63) Cross-sectional	North-Central ART clinics	NR - verify	Screening/vaccination	61.5% of WLWH had never been screened for cervical cancer.	Mod
Non-Hodgkin lymphoma					
Silas et al. (69) Retrospective cohort	JUTH, Jos; 2005-2015	40 (8 HIV+, 32 HIV-)	HIV-associated lymphoma	HIV ~3x mortality; 2-yr survival 30% vs 74% (HIV+ vs -).	Low
Oluwasola et al. (38) 15-yr review	UCH, Ibadan; 1991-2005	NR - verify	Lymphoma	Decline in high-grade NHL (79.1%->21.1%) and Burkitt lymphoma (45.8%->13.6%).	Mod
Salawu et al. (37) Retrospective	OAUTH, Ile-Ife (SW)	NR - verify	AIDS-related lymphoma	NHL predominates; AIDS-related lymphoma an emerging phenomenon.	Mod
Bolarinwa et al. (39) Retrospective	OAUTH, Ile-Ife (SW)	NR - verify	AIDS-related lymphoma	Characterization of AIDS-related lymphomas in Nigeria.	Mod
Non-AIDS-defining malignancies (NADMs)					
Okeke et al. (32) Prospective cohort	JUTH, Jos; 2015-2017	101 HCC (10 HIV+, 91 HIV-)	Hepatocellular carcinoma	HIV+ shorter survival (3-mo 22% vs 47%); high early mortality.	Mod
Davwar et al. (33) Prospective cohort	JUTH & LUTH; 2018-2021	213 HCC (36 HIV+ [16.9%], 177 HIV-)	Hepatocellular carcinoma	78% present at BCLC stage C/D; HIV+ younger; limited access to curative therapy.	Low
Zheng et al. (31) Biomarker study	Nigerian HIV+ cohort	NR - verify	HCC risk (cfDNA methylation)	Circulating cell-free DNA methylation markers for HCC risk prediction.	Low
Ibanga et al. (40) Case series	Tertiary eye center, Calabar	NR - verify	Conjunctival SCC	65.6% HIV+; predominantly women in their 30s; no radiotherapy/chemotherapy access.	Mod
Fasina (41) 10-yr review	Ibadan (SW)	NR - verify	Ocular surface neoplasia	50% HIV+; mean age 48.2 yr; >one-third invasive carcinoma.	Mod
Cross-cutting: registry linkage & meta-analyses					
Akarolo-Anthony et al. (23) Registry linkage	IHVN + Abuja registry; 2009-2012	17,826 PLHIV (39 cancers)	AIDS-Cancer Match	NADCs most common; SIRs mostly not elevated - likely under-ascertainment.	Low
Stelzle et al. (57) Systematic review & meta-analysis	Global (24 studies)	236,127 WLWH	ICC risk in WLWH	~6-fold increased ICC risk in WLWH (RR 6.07, 95% CI 4.40-8.37).	Low
Shiels et al. (70) Meta-analysis	Multinational	Pooled - verify	NADC incidence	Elevated incidence of (esp. infection-related) NADCs in PLHIV.	Low
Yuan et al. (71) Systematic review & meta-analysis	Global (46 studies)	Pooled - verify	NADC incidence & mortality	Higher incidence and mortality of NADCs among PLHIV across regions.	Low

Grouped by malignancy. Reference numbers follow the reference list in this document.

RoB, risk of bias (adapted Newcastle-Ottawa domains). “NR - verify” = sample size not extractable from the manuscript text; confirm from source before submission. Abbreviations: PLHIV, people living with HIV; WLWH, women living with HIV; ADM, AIDS-defining malignancy; NADC/NADM, non-AIDS-defining cancer/malignancy; ICC, invasive cervical cancer; KS, Kaposi sarcoma; NHL, non-Hodgkin lymphoma; HCC, hepatocellular carcinoma; SCC, squamous cell carcinoma; HR-HPV, high-risk HPV; PY, person-years; OS, overall survival; SIR, standardized incidence ratio; F, female. Centers: JUTH, Jos UTH; LUTH, Lagos UTH; UCH, University College Hospital, Ibadan; OAUTH, Obafemi Awolowo UTH; ABUTH, Ahmadu Bello UTH; UBTH, University of Benin TH; NIMR, Nigerian Institute of Medical Research; IHVN, Institute of Human Virology Nigeria.

## 3 Results

### 3.1 Overview of included studies

Our search identified 847 records across all databases and sources. After removing duplicates, screening titles and abstracts, 127 full-text articles were reviewed, of which 73 met the inclusion criteria and were included in this narrative synthesis. Most studies were cross-sectional and retrospective (n = 38; 52.1%), followed by cohort studies (n = 12; 16.4%), case series (n = 10; 13.7%), cancer registry reports (n=5; 6.8%), policy documents and grey literature (n=5; 6.8%), and systematic reviews (n=3; 4.2%). See **Figure 1** for a PRISMA flow chart of screening and study selection. Most studies were conducted in southwestern Nigeria (n = 31; 42%), mainly in Lagos and Ibadan. Northern regions, including Jos and Kano, contributed fewer studies (n = 11; 15%), with the rest from other parts of the country. This distribution highlights important geographic gaps in the available evidence. Although southwestern centers contributed the most studies, the most detailed longitudinal evidence, including Kaposi sarcoma incidence trends, lymphoma survival, and hepatocellular carcinoma cohorts, comes from north-central (Jos/JUTH) and affiliated multi-institutional collaborations; the geographic distribution of study volume therefore differs from that of analytic depth. The records showed that publication timing was concentrated between 2008 and 2016, with notable growth in registry-based studies after 2012 and renewed research activity from 2020 to 2024. Most studies were conducted at major urban teaching hospitals, especially in Jos, Lagos, Ibadan, Kano, Zaria, and Enugu, which may introduce selection bias toward tertiary care populations and more advanced disease presentations. The bulk of the population with HIVAM in Nigeria are managed at tertiary centers, with a large percentage of cases coming from rural facility referrals.

**Figure 1:**
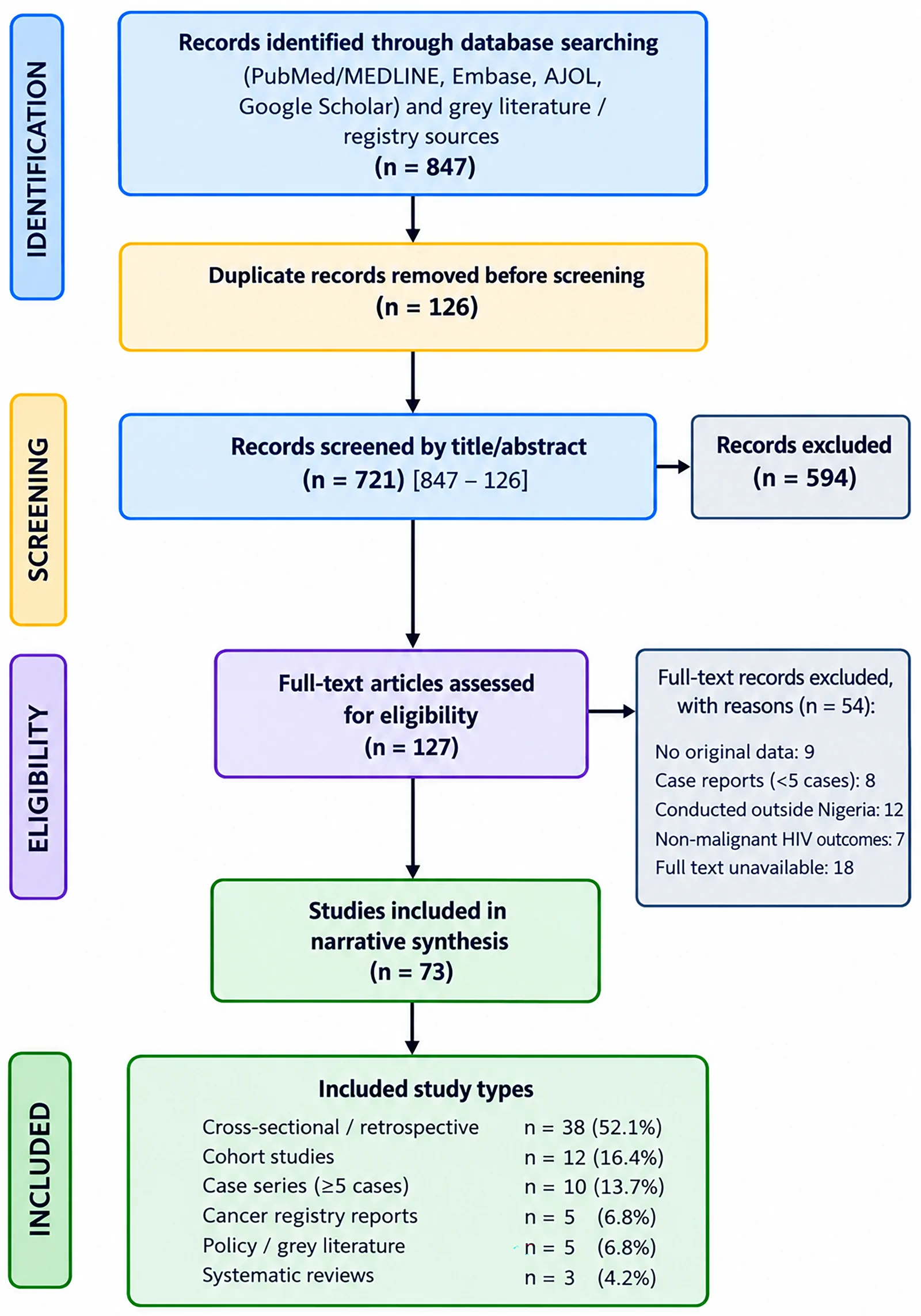
PRISMA-style study-selection flow.

### 3.2 Burden of HIV-associated malignancies in Nigeria

#### 3.2.1 Overall magnitude

Accurate quantification of HIVAM burden in Nigeria remains constrained by incomplete population-based cancer registration and limited linkage between HIV and cancer datasets (13, 14, 16, 17, 21). Most available estimates, therefore, come from hospital-based studies and a small number of registries, likely underestimating the true population burden. The NSCR, established in 2009, supports population-based registries in Ibadan, Abuja, and Calabar, but HIV status ascertainment within these registries has been inconsistent, limiting burden estimates (17, 21). A multi-institution hospital registry study across 11 tertiary hospitals reported 6,484 cancer cases (2009–2010), with HIV status variably recorded (17). In contrast, the Nigerian AIDS-Cancer Match Study (Institute of Human Virology Nigeria) provided a more systematic assessment by linking HIV treatment records with cancer registry data among 17,826 HIV-positive persons followed from 2005–2012 using matching algorithms, representing one of the few national-scale HIV–cancer linkage efforts in West Africa (23).

#### 3.2.2 Temporal trends

Available data from registries and hospital series suggest evolving patterns. IBCR and LUTH registry summaries suggest: (i) a modest decline in KS cases since widespread ART rollout; (ii) stable or increasing numbers of cervical cancer cases; and (iii) rising detection of breast and prostate cancer, likely reflecting both population aging and improved detection (13, 17, 21). JUTH registry data indicate a particularly high burden of KS, including pediatric KS, in the north-central region, with decreasing incidence post-ART scale-up but persistent late-stage presentation (19).

#### 3.2.3 Evidence from national programs and policy documents

NACA surveillance reports and FMOH cancer control documents acknowledge HIVAMs as an emerging national concern but highlight the lack of integrated HIV–cancer data systems (3, 13). Nigeria recorded an estimated 127,763 new cancer cases in 2022 (GLOBOCAN 2022), with breast and cervical cancer the leading malignancies among women, including women living with HIV (WLWH), based on national cancer control planning data and GLOBOCAN 2022 estimates (1, 13, 24). However, HIV status is not systematically captured, and cancer-related mortality among PLHIV is likely underreported.

Overall, in major Nigerian centers, ADMs, especially ICC, KS, and NHL, were the most common cancers documented among PLHIV (13–25). ICC consistently appears as the leading cancer among WLWH (25–30). There is geographical variation, with northern centers such as Jos and Kano reporting higher KS rates, while ICC is more common in southwestern facilities such as Ibadan and Lagos (13–24). NADMs, including HCC, Hodgkin lymphoma, breast cancer, and conjunctival squamous cell carcinoma, were identified but were much less common, reflecting both survival-related epidemiological changes and limited diagnostic resources (31–41).

### 3.3 AIDS-defining malignancies

#### 3.3.1 Kaposi sarcoma

KS is the most extensively reported HIVAM in Nigeria, with 15–20 publications identified (42–55). Hospital-based studies consistently show that KS is the leading cancer among PLHIV in Nigeria. Several hospital-based studies from JUTH have recorded a significant KS burden among PLHIV over cohorts from 2006 to 2023 (43). Additionally, a 14-year retrospective study at LUTH identified KS as a leading ADM (46). Regional studies from Benin City, Calabar, Sokoto, and Maiduguri repeatedly identified KS as the most common malignancy in PLHIV, though regional incidence rates differed (47–51).

Clinical patterns indicate that KS predominantly affects males, with male-to-female ratios ranging from 1.8:1 to 3.2:1 (42–51). The age at presentation is generally young, with a mean of 32 to 38 years, lower than in classical endemic KS (42–51). A JUTH study (Agaba et al.) reported poor treatment outcomes in the pre-ART era, with marked survival improvement following widespread ART scale-up (45). Nonetheless, KS associated with immune reconstitution inflammatory syndrome (IRIS) remains a clinical challenge, particularly in newly diagnosed HIV patients starting ART with severe immunosuppression (52). Currently, the incidence of KS is decreasing, and ICC rates have remained stable over time (43–55). A retrospective study from JUTH, one of Nigeria’s largest HIV treatment centers, found that KS decreased from 2.53 to 1.58 cases per 1,000 person-years between 2006 and 2016 (43). Similar patterns are observed in other clinical settings, where ART appears to reduce KS more effectively than it reduces ICC (54, 55). Registry data from IBCR and LUTH show modest declines in KS, stable or increasing ICC cases, and rising detections of breast and prostate cancers, likely due to aging populations and improved diagnostics (21, 56).

#### 3.3.2 Invasive cervical cancer

ICC is the most common cancer among Nigerian WLWH and was the second most frequently studied HIVAM, with 12–18 publications identified in this review (26–31, 57–62). Evidence from a systematic review and meta-analysis of 24 studies across four continents (n = 236,127 WLWH) reported a sixfold increased risk of invasive cervical cancer for WLWH(RR 6.07, 95% CI 4.40–8.37) (56, 57). Studies consistently show that WLWH have increased high-risk HPV genotype diversity and experienced rapid progression to cervical intraepithelial neoplasia. ICC tends to be diagnosed at a younger age with more advanced disease compared to HIV-negative women, due to accelerated HPV carcinogenesis, immunosuppression, HIV related inflammation, and limited cervical cancer screening (26–31). A large multi-institutional cohort from Jos and Lagos reported that HIV-positive women with histologically confirmed ICC were diagnosed about ten years earlier, had significantly lower 12-month survival rates, and showed lower treatment completion compared to HIV-negative women (26). These outcomes are associated with late-stage presentation, severe immunosuppression, and treatment interruptions, highlighting systemic barriers and socioeconomic challenges influencing disease progression. Across different centers, ICC in WLWH was characterized by multifocal lesions, rapid tumor invasion, and higher recurrence rates, while baseline anemia and poor performance status were also more common among HIV-positive women, contributing to worse prognosis (57–61).

Despite the higher risk of ICC in WLWH, cervical screening coverage in Nigeria remains very low. A recent cross-sectional study among women attending ART clinics in North-Central Nigeria reported that 61.5% had never been screened for cervical cancer, highlighting poor integration of screening into HIV care, while HPV vaccination coverage remains extremely limited in routine practice (63). Data from facility-based studies from Abuja, Jos, and Lagos consistently demonstrate low cervical cancer screening uptake among WLWH, with most reports indicating that fewer than 20–30% of eligible women have ever been screened or received regular screening, reflecting weak integration of cervical cancer prevention into HIV care services (64–66). Barriers such as low awareness, stigma, weak healthcare infrastructure, shortages of trained staff, and, most importantly, weak integration of cervical cancer prevention services into routine HIV care result in persistently low screening uptake across settings. The World Health Organization (WHO) recommends single-visit “screen-and-treat” approaches using thermal ablation to improve treatment uptake and treatment completion, particularly in low-resource settings, rather than multi-visit approaches with cytology or colposcopy (67). To address these challenges, the National Strategic Plan for Prevention and Control of Cancer of the Cervix in Nigeria (2023–2027) prioritizes a comprehensive approach that includes expanding cervical cancer screening and strengthening service integration, with WLWH identified as a high-risk group requiring more frequent screening within the health system (68).

These efforts highlight the urgent need to expand integrated screening within ART programs to lower cervical cancer risks among WLWH. Nigeria introduced HPV vaccination nationally in October 2023, targeting girls aged 9–14 years with a single-dose vaccine [NPHCDA/NGI-TAG, 2023]. However, this adolescent-focused program does not specifically address adult WLWH, who remain at increased risk of persistent infection with oncogenic HPV types not covered by the vaccine formulation, including HPV35 (30). Continued surveillance, intensified screening, and consideration of broader-valency vaccines may therefore be important components of cervical cancer prevention among WLWH in Nigeria.

#### 3.3.3 Non-Hodgkin lymphoma

Evidence from Nigerian hospital-based studies indicates that HIVAMs, particularly lymphomas, contribute significantly to cancer morbidity and mortality, although reported prevalence remains lower than in high-income settings (69). NHL, which is the third most common ADM affecting PLHIV, is the most prevalent among all lymphomas (37–39, 69). Diffuse large B-cell lymphoma is the most common HIV-associated NHL in Nigeria (38). Patients often present with advanced disease, extranodal involvement, B symptoms, and bone marrow infiltration (37–39). Other cancers identified in PLHIV include squamous cell carcinomas, Burkitt lymphoma, and solid tumors, but data on non-AIDS-defining cancers are limited (37–39, 69).

A 15-year retrospective review at University College Hospital, Ibadan, documented an overall decline in lymphoma cases, with high-grade NHL falling from 79.1% to 21.1% and Burkitt’s lymphoma from 45.8% to 13.6% between 1991 and 2005 (38). Notably, the findings suggest that the rising HIV epidemic in Nigeria did not lead to proportional increases in lymphoma cases. Most HIV-associated lymphomas did not display features typical of AIDS-related subtypes like CNS or primary effusion lymphoma (38). Similarly, a retrospective review at Obafemi Awolowo University Teaching Hospital (1993–2008) identified HIV seropositivity in only 2.3% of lymphoma patients, with NHL predominating among HIV-positive cases (37). However, despite established regional endemicity, no AIDS-associated Burkitt lymphoma was identified in this study, further underscoring distinct epidemiologic patterns of HIV-related lymphoma in Nigeria (37). Affected patients consistently presented at advanced disease stages, with rapid clinical deterioration and markedly short survival.

These observations were reinforced by a retrospective cohort study at JUTH (2005–2015), wherein HIV infection was associated with a threefold increased risk of death following lymphoma diagnosis, and two-year survival rates were markedly lower among HIV-positive patients compared to HIV-negative counterparts (30% vs. 74%) (69). Late-stage presentation emerged as the principal predictor of mortality, reflecting critical systemic delays in diagnosis and treatment. Collectively, these findings highlight the urgent need for expanded ART coverage, early cancer detection strategies, and subsidized chemotherapy to reverse these unfavorable trends across Nigerian healthcare settings.

### 3.4 Non-AIDS-defining malignancies

The spectrum of NADMs among Nigerian PLHIV is less well characterized than ADMs, reflecting both a relatively recent epidemiological transition and limited surveillance (1, 4, 5, 23). Although NADMs are less common, they are increasingly being reported as an important contributor to morbidity and mortality among PLHIV who live longer on ART (70, 71). Early meta-analyses from predominantly high-income settings demonstrated consistently elevated risks of a broad spectrum of NADMs in PLHIV, particularly infection-related malignancies such as HPV–associated cancers, liver cancer, and Hodgkin lymphoma, underscoring the central role of HIV-related immune dysfunction and inflammation (70). Subsequent pooled analyses and more recent global evidence consistently show that HIV infection is associated with increased incidence and mortality of NADMs, with the greatest risks observed among individuals with advanced immunodeficiency (71). This systematic review and meta-analysis of 46 cohort and registry-linkage studies further demonstrates that all infection-related NADMs and nearly half of non-infection-related cancers occur at higher rates among people living with HIV across diverse regions and income settings. Persistently elevated mortality has been reported for cancers such as anal cancer, Hodgkin lymphoma, liver cancer, lung cancer, and melanoma (71).

However, data remains limited. A population-based registry-linkage study from Abuja found that non-AIDS-defining cancers accounted for most cancers among PLHIV, including breast, liver, anal, prostate, ovarian, and skin cancers, although standardized incidence ratios for most NADCs were not significantly elevated compared with the general population (23). This likely reflects underdiagnosis, incomplete cancer data, and limited registry coverage rather than a true lack of risk (23).

An increasing number of hospital-based studies report high rates of liver cancer, ocular surface squamous neoplasia (OSSN), and conjunctival squamous cell carcinoma (SCC) among HIV-positive adults, who are often younger and disproportionately women (23, 31–33, 40, 41). At a tertiary eye center in Calabar, 65.6% of conjunctival SCC cases were HIV-positive, mainly affecting women in their 30s, who had no access to radiotherapy or chemotherapy (40). In southwestern Nigeria, a 10-year review of OSSN found that 50% of patients were HIV-positive, with a mean age of 48.2 years, and more than one-third showed invasive carcinoma (41). In a prospective cohort from Jos and Lagos, 16.9% of HCC cases were HIV-positive, and the median age was 43.9 years (31–33). Most presented at advanced stages (Barcelona C or D) and lacked access to curative therapy (32). Anal cancer, strongly associated with HPV infection, is an emerging concern among PLHIV, particularly men who have sex with men (MSM) (72). Data from Nigeria are extremely limited, reflecting both possible low detection and social/legal barriers to MSM-focused research and care (73).

Other reported malignancies among Nigerian PLHIV include lung, breast, and prostate cancers, as well as leukemias (23). However, current data do not provide a definitive measure of increased risk. Often, these cancers may be attributed to aging rather than direct links to HIV. Notably, an emerging pattern of early-onset, aggressive NADMs in young and middle-aged adults is exacerbated by delayed diagnosis and poor integration of HIV-oncology services, underscoring the urgent need for well-designed comparative studies. The role of accelerating aging in several of these earlier occurring cancers is also a subject of keen interest.

### 3.5 Challenges in managing HIV-associated malignancies

#### 3.5.1 Surveillance and data systems

The lack of integrated HIV-cancer surveillance is a major obstacle to understanding and addressing HIVAM burden in Nigeria. HIV status is often not included in most cancer registries, and cancer diagnoses are rarely recorded in HIV treatment databases. This disconnect prevents accurate estimation of burden, hampers epidemiological research, and obstructs the development of evidence-based policies. The Nigerian AIDS-Cancer Match Study showed that linking registries is feasible and valuable but also revealed significant technical, logistical, and resource challenges (23). Expanding these efforts requires investing in data infrastructure, implementing standardized coding systems, establishing unique patient identifiers that safeguard privacy, and developing technical capacity for probabilistic matching algorithms. Additionally, since most research is concentrated in southwestern Nigeria, patterns in northern, southeastern, and other regions remain poorly understood, likely obscuring important variations in cancer burden, types, and outcomes across Nigeria’s diverse ecological and cultural zones.

#### 3.5.2 Diagnostic capacity

Diagnostic limitations significantly impede accurate cancer identification and staging in Nigeria. Histopathology services are mainly centralized in tertiary centers, with ongoing shortages of trained pathologists, limited immunohistochemistry capabilities, and almost no access to molecular diagnostics at most facilities (2, 13, 14, 16). For lymphomas, precise subtyping depends on immunophenotyping and molecular studies that are rarely available, causing diagnostic delays and misclassification. Likewise, cervical cancer screening remains insufficient, with fewer than 25% of ART clinics providing screening services (58). Advanced imaging techniques, such as CT and MRI for cancer staging, are only accessible in major urban centers and often come with high out-of-pocket costs. These diagnostic deficiencies lead to late-stage diagnoses, fewer treatment options, and poorer outcomes, particularly impacting PLHIV who may have more aggressive disease biology.

#### 3.5.3 Treatment access and affordability

Access to cancer treatment in Nigeria faces severe challenges due to limited infrastructure, inadequate human resources, and major financial barriers. Radiotherapy services are available in fewer than ten facilities nationwide, with most machines often non-operational because of maintenance issues (13, 14). Chemotherapy access is also limited, with frequent shortages of essential drugs and costs that are prohibitive for most patients. For PLHIV, these barriers are worsened by the need for coordinated HIV-oncology care that addresses drug interactions, manages treatment-related immunosuppression, and ensures ART adherence during cancer treatment. The lack of universal health coverage means most cancer care expenses are paid out-of-pocket, leading to catastrophic health costs, treatment abandonment, and significant financial hardship for families. Studies from Jos and Lagos consistently show high rates of treatment refusal, or abandonment due to cost, especially among PLHIV, who face additional challenges because of their ongoing need for HIV care services.

#### 3.5.4 Integration of HIV and cancer services

The vertical structure of HIV and cancer services in Nigeria creates significant inefficiencies and missed opportunities for prevention and early detection. ART programs generally lack integration with cancer screening, diagnosis, and treatment services, despite PLHIV being a high-risk group that would benefit from systematic surveillance (3, 14, 16, 24). Conversely, cancer centers rarely have the expertise or infrastructure to manage HIV treatment, resulting in fragmented care for patients with both conditions. This lack of integration leads to poor screening uptake, delayed cancer diagnoses, suboptimal treatment coordination, and inadequate survivorship care. Successful integration requires the co-location of services, cross-training of healthcare workers, shared medical records, joint clinical protocols, and coordinated referral pathways, all of which are largely absent in the current Nigerian health system. The National Strategic Plan for Cervical Cancer Prevention (2023-2027) is an important step toward integration, but it requires substantial investment in implementation to achieve its goals (68).

#### 3.5.5 Patient-level barriers

Beyond health system limitations, patient-level factors significantly affect HIVAM outcomes in Nigeria. Low cancer awareness, especially about early warning signs and screening, leads to late presentation across all cancer types (17, 23). HIV-related stigma adds more barriers, with patients often hesitant to disclose their status or seek care in facilities where they might face discrimination. Financial challenges limit not only access to treatment but also to diagnostic tests, supportive care, and transportation to specialized centers. Cultural beliefs and the preference for alternative therapies can delay patients from seeking conventional healthcare. For women with cervical cancer, patriarchal structures may restrict autonomy in healthcare decisions, with some needing spousal permission to get treatment (23, 26). Geographic barriers are especially severe in rural areas, where patients might travel hundreds of kilometers to access cancer diagnosis and treatment services, creating logistical and financial hardships that cause delays or abandonment of treatment.

## 4 Discussion

This narrative review consolidates existing evidence on the burden, patterns, and challenges of HIVAMs in Nigeria. It highlights a landscape characterized by a significant yet under-measured cancer burden, diverse epidemiologic patterns across regions and care settings, and health system constraints that delay diagnosis and restrict effective treatment. Nigeria’s capacity to reduce HIVAM mortality will rely on strengthening HIV–cancer surveillance linkages, expanding integrated prevention efforts (especially for cervical cancer), and enhancing diagnostic and treatment capabilities throughout the entire care continuum.

### 4.1 Burden and epidemiologic patterns

ADMs remain dominant, especially ICC, KS, and NHL, consistent with the biology of immunosuppression and oncogenic viral co-infections. ICC is the clearest high-burden priority: a large meta-analysis (24 studies; 236,127 WLWH) estimated that WLWH have about a six-fold higher risk of cervical cancer than HIV-negative women (57). This increased risk is biologically plausible because HIV promotes the acquisition and persistence of high-risk HPV and accelerates the progression of cervical precancer, especially with low CD4 counts and limited screening access (74). Another important contribution is inflammation. The cellular senescence promoted by HIV produces a hypersecretory state - “senescence-associated secretory phenotype” which, through a pro-inflammatory microenvironment and signaling pathways, can promote tumor progression and cancer cellproliferation (75).

Across sub-Saharan Africa, pooled data show that HPV infection is much more common in WLWH than in HIV-negative women (e.g., 53.6% vs 26.5% in a 20-year systematic review) (76). Although Nigeria-specific HPV prevalence estimates vary by study setting, this regional pattern supports the expectation of a significant HPV-related burden among Nigerian WLWH and highlights the urgent need to integrate HPV vaccination and cervical screening within ART services (30).

For Kaposi sarcoma, evidence from Nigeria-specific cohorts suggests a decline in incidence alongside the expansion of ART policies: in a retrospective cohort covering 2006–2016, the crude KS incidence decreased from 2.53 to 1.58 per 1,000 person-years during periods of increased ART eligibility (43). However, hospital-based data may under-represent community burden and could be biased toward late referrals; ongoing reports of advanced cases highlight persistent diagnostic delays and incomplete HIV care engagement, especially among men and underserved populations.

The review indicates that NADMs are becoming more prominent as survival improves with antiretroviral therapy and the aging population of people living with HIV. This trend does not imply a uniform increase in risk across all cancer types; however, it underscores the need for health systems to strengthen capacity for early detection and management, particularly in Nigeria, where cancers often present at advanced stages. Importantly, the development of NADMs in PLHIV is driven by a combination of multiple factors, including aging (and accelerated aging), chronic immune dysregulation and inflammation, and exposure to oncogenic infections and carcinogens such as hepatitis B virus, hepatitis C virus, human papillomavirus, Epstein Barr virus, aflatoxin, and tobacco use. (71).

A key NADM signal in Nigeria is HCC, where HIV intersects with HBV/HCV co-infection and late-stage presentation. Nigerian data indicate substantial HBV/HCV co-infection prevalence among PLHIV, and HCC cohorts highlight HBV/HCV as major correlates of HCC burden and outcomes (31–33). These findings strengthen the case for integrating HBV vaccination, HBV/HCV screening, and treatment into HIV programs as a cancer prevention strategy.

For OSSN, evidence from Africa consistently links rising incidence with HIV and indicates a trend toward younger age at presentation and a significant disease burden (40, 41). A Nigeria-focused synthesis should consider OSSN as a meaningful yet under-detected HIV-associated cancer phenotype that needs improved referral pathways and pathology confirmation.

### 4.2 Surveillance and measurement constraints

A central interpretation issue is that Nigeria’s HIVAM burden is likely underestimated because most evidence comes from hospital cohorts and fragmented registries with incomplete HIV status documentation. The most compelling Nigerian example of feasible linkage is the Nigerian AIDS–Cancer Match Study, which probabilistically connected an HIV cohort (IHVN) with the Abuja Cancer Registry (2009–2012) (23). Among 17,826 PLHIV (median age 33 years; 59% women), 39 cancers were identified, demonstrating both feasibility and the potential for under-ascertainment without linkage (23). This supports the argument that Nigeria should establish HIV–cancer linkage within the National System of Cancer Registries and major HIV treatment databases, with governance frameworks that safeguard privacy while allowing public health monitoring.

### 4.3 Health system imperatives: diagnosis, treatment, and service integration

Diagnosis remains a bottleneck. Delays stem from workforce shortages (pathologists, oncology nurses, radiation oncologists), uneven geographic distribution of services, and weak specimen referral and quality assurance systems. Increasing pathology capacity requires building training pipelines, maintaining equipment, standardizing reporting, and implementing practical telepathology models to expand specialist access beyond tertiary centers.

For cervical cancer prevention within HIV care, Nigeria has developed a program experience using VIA-based screening (Visual inspection with Acetic Acid) and thermal ablation, but retention throughout the cascade remains insufficient. A large program screening WLWH reported treatment with thermal ablation among VIA-positive clients and highlighted gaps in post-treatment follow-up, reinforcing that “screen-and-treat” success depends not only on screening tools but also on workflow design, supplies, tracking, and adherence support (77). These realities align with Nigeria’s policy direction, calling for scaling up cervical cancer prevention and integrating approaches (68).

Treatment access is a major determinant of outcomes. Radiotherapy shortages and frequent treatment interruptions have been documented in Nigerian oncology care, mainly caused by equipment limitations, maintenance issues, and power instability (78). These challenges are especially critical for ICC and other potentially curable cancers. As a result, improving cancer care involves (i) increasing radiotherapy capacity and uptime, (ii) ensuring availability of chemotherapy and supportive care, (iii) establishing multidisciplinary tumor boards (including HIV clinicians), and (iv) implementing financing reforms to lower catastrophic out-of-pocket expenses.

Integration is a key strategic opportunity. Nigeria’s HIV platform is extensive, and recent strategic documents show that many Nigerians living with HIV are on ART, providing a foundation for cancer screening, triage, referral, and long-term follow-up (79). Integration should focus on high-yield services first: cervical screening and ablation, HBV vaccination and testing/treatment for HBV and HCV, and symptom-triggered pathways for lymphoma and KS.

### 4.4 Research priorities

1. Population-based risk quantification through linkage. Nigeria needs multi-site registry linkage studies expanding beyond Abuja to estimate standardized incidence ratios and trends by ART era, CD4/viral suppression status, sex, and age. The Abuja linkage study demonstrates feasibility and can be scaled (23).
2. Prospective HIV cohorts with cancer endpoints. Follow-up designs should include ART timing, viral suppression, immunologic recovery, and co-infections to explain heterogeneity in cancer risk and stage at diagnosis.
3. Implementation of science for integrated prevention. Studies should compare models for cervical screening delivery within ART clinics (VIA vs HPV testing triage where feasible; appointment systems; community follow-up), measure retention, and cost-effectiveness (77).
4. Cancer-specific etiologic investigations in Nigeria. Priority gaps include lymphoma subtyping (and EBV association), liver cancer pathways (HBV/HCV/aflatoxin and timing of antiviral therapy), and improved understanding of OSSN burden in PLHIV (33).
5. Understudied populations. Research on anal HPV/anal cancer risk in key populations and pediatric/adolescent HIVAM surveillance is needed, even in challenging legal/social contexts, because program blind spots lead to preventable mortality.

### 4.5 Policy and programmatic recommendations (expanded and made more “actionable”)

1. Establish a national HIV–cancer coordination mechanism (FMOH/NACA + NICRAT + NSCR + major implementing partners) to standardize HIV status capture in cancer settings, define minimum oncology datasets in HIV clinics, and oversee linkage governance (68).
2. Scale integrated cervical cancer prevention in HIV care with a practical cascade mindset: screening coverage targets must be matched with ablation capacity, referral pathways for suspected cancer, and tracking systems to minimize loss to follow-up. Evidence from Nigerian programs shows that follow-up can be a major failure point even after expanding screening (77).
3. Integrate HBV vaccination and HBV/HCV screening and treatment into HIV clinics as a form of cancer prevention, emphasizing HCC risk reduction and decreasing liver disease mortality (33).
4. Invest in pathology and radiotherapy as shared infrastructure that benefits both PLHIV and the general population. Access to radiotherapy and its continuity are well-documented challenges in Nigeria; addressing uptime, maintenance, and workforce capacity should be a national cancer control priority (78).
5. Develop high-throughput molecular diagnostic laboratories for HIV-associated cancers. Strategic investment in regional high-throughput molecular diagnostic laboratories is necessary to support early detection, risk stratification, and surveillance of HIV-related malignancies. Incorporating services such as HPV genotyping, viral hepatitis testing, molecular lymphoma subtyping, and epigenetic biomarker assays into existing academic and ART-linked laboratories would enable scalable precision diagnostics, strengthen cancer surveillance, and facilitate translation of research into routine HIV care.
6. Utilize AI and digital health technologies where they resolve a bottleneck. High-impact applications include automated appointment scheduling and defaulter tracking for screening follow-up; electronic cancer synoptic reporting; probabilistic linkage algorithms for HIV–cancer matching (building on the Abuja experience); and decision support for symptom-based referral triage informed by CD4/viral load and basic labs.

### 4.6 Strengths and limitations

This review highlights strengths as one of the most comprehensive on HIV-related cancers in Nigeria, combining data across regions, study types, and sources like cancer registries and policy documents to provide a broad, policy-relevant view of epidemiology, health system issues, and research priorities. Its integration of clinical, public health, and implementation insights makes it useful for policymakers, program managers, and researchers at the HIV-cancer interface.

However, limitations include reliance on hospital-based, retrospective studies that are prone to bias and do not represent all PLHIV, especially those not engaged in care. The focus on southwestern Nigeria limits regional understanding, with northern and southeastern areas being underrepresented. Incomplete HIV status data and inconsistent cancer documentation hinder accurate assessment of the burden, as does limited diagnostic capacity, particularly for lymphomas. Heterogeneity among studies prevents quantitative analysis, and the narrative approach lacks formal bias assessment. In addition, a substantial proportion of the Nigeria-specific primary data synthesized here (e.g., Kaposi sarcoma trends, cervical cancer epidemiology, lymphoma survival, and hepatocellular carcinoma) originates from the Jos/JUTH and affiliated Lagos–Miami/Northwestern collaborative networks, several members of which are co-authors; while this reflects these groups’ sustained output in HIV–cancer research in Nigeria, it may introduce selection and citation bias and over-represent north-central data relative to the national picture. Despite these challenges, the consistent patterns across studies emphasize the need for more rigorous, population-based research.

## 5 Conclusion

HIV-associated malignancies present a significant and likely under-recognized public health challenge in Nigeria. While notable progress has been achieved through expanding ART, AIDS-defining cancers, particularly invasive cervical cancer, Kaposi’s sarcoma, and non-Hodgkin lymphoma, continue to cause substantial illness and death among people living with HIV. At the same time, the increasing incidence of non-AIDS-defining malignancies reflects better survival rates, an aging population, and the cumulative effects of accelerated aging, chronic immune dysregulation, and oncogenic virus co-infection.

The evidence summarized in this review shows that Nigeria’s burden of HIV-associated malignancies is influenced as much by health system limitations as by biological risk factors. Fragmented surveillance systems, delays in diagnosis, insufficient pathology and radiotherapy capacity, high out-of-pocket costs for treatment, and weak integration between HIV and oncology services collectively result in late-stage diagnoses and poor outcomes. However, these challenges also offer a major opportunity: Nigeria’s extensive HIV care infrastructure provides a scalable platform for integrated cancer prevention, early detection, referral, and ongoing follow-up.

Reducing the impact of HIV-associated malignancies in Nigeria requires coordinated efforts across surveillance, prevention, diagnosis, treatment, and research. Key actions include developing sustainable linkage systems between HIV and cancer registries, expanding integrated cervical cancer screening and HPV vaccination within ART programs, strengthening pathology and oncology services, and incorporating hepatitis B vaccination and viral hepatitis management into routine HIV care. Investing in implementation research, training the health workforce, and enhancing health information systems, including the appropriate use of digital tools and artificial intelligence for surveillance and care coordination, will be essential to guide efficient, equitable, and contextually appropriate interventions.

Without intentional integration of cancer control into HIV programs, preventable cancer-related deaths among people living with HIV will continue to undermine the progress made through ART. In contrast, a coordinated, evidence-based approach has the potential to significantly reduce morbidity and mortality and promote more equitable outcomes for this growing and vulnerable population.

## Group members of TAGCReC

Andrea Oludolapo Akinjo^1^, Asmau Aisha^2^, Dauda Abubakar Katagum^3^, Abubakar Hussaini Nuhu^4^, Sunday E. Achanya^3^, Emmanuel Onyilo Agbogo^3^, Fatima Munaya Isyaku^5^, Louis Ndubuisi Ukaegbu^6^, Uchenna Simon Ezenkwa^7^, Damilola Odusolu^8^, Faith Akpe Ikpan^8^, Aisha Mustapha^9^, Maradona Isikhumen^10^, Michael C. Azikem^10^, Laura Adetunji^10^, Michael C. Ezeanochie^10^, Victory Akhidue^11^, Victor Ekanem^11^, Patience Odusolu^12^, Ayomidipupo Shobakin^13^, Chigbo Jerome Ikechukwu^14^, John Damter^14^, Golit William Nengak^14^

1College of Medicine/Lagos University Teaching Hospital, University of Lagos State, Nigeria

2Katsina state University, Katsina State, Nigeria

3Department of Obstetrics and Gynecology, Federal University of Health Sciences, Bauchi State, Nigeria

4Department of Histopathology Federal University of Health Sciences, Bauchi State, Nigeria

5Department of Histopathology, Federal University of Health Sciences Teaching Hospital Azare, Bauchi State, Nigeria

6Department of Haematology and Blood Transfusion FUHSTHA, Bauchi State, Nigeria

7Department of Histopathology Federal University of Health Sciences Azare, Bauchi State, Nigeria

8University of Calabar Teaching Hospital, Cross-River State, Nigeria

9ABUTH, Zaria, Kaduna State, Nigeria10Department of Obstetrics and Gynaecology, University of Benin Teaching Hospital, Edo State, Nigeria

11Department of Morbid Anatomy and Histopathology, University of Benin Teaching Hospital, Edo State, Nigeria

12University of Calabar, Calabar Nigeria

13Department of Biochemistry, College of Medicine/ Lagos University Teaching Hospital, Idiaraba, Lagos, Nigeria

14Department of Obstetrics and Gynecology, Plateau State Specialist Hospital, Jos, Nigeria

## References

[1] GBD 2021 HIV Collaborators . Global, regional, and national burden of HIV/AIDS, 1990-2021, and forecasts to 2050, for 204 countries and territories: the Global Burden of Disease Study 2021. Lancet HIV. (2024) 11:e807–22. doi: 10.1016/S2352-3018(24)00212-1 39608393 PMC11612058

[2] Onovo AA Adeyemi A Onime D Kalnoky M Kagniniwa B Dessie M . Estimation of HIV prevalence and burden in Nigeria: a Bayesian predictive modelling study. EClinicalMedicine. (2023) 62:102098. doi: 10.1016/j.eclinm.2023.102098 37538543 PMC10393599

[3] National Agency for the Control of AIDS . Improving Nigeria’s HIV fight: The transformative power of state ownership (2023). Available online at: https://naca.gov.ng/improving-nigerias-hiv-fight-the-transformative-power-of-state-ownership/ (Accessed August 4, 2026).

[4] Bashorun A Nguku P Kawu I Ngige E Ogundiran A Sabitu K . A description of HIV prevalence trends in Nigeria from 2001 to 2010: what is the progress, where is the problem? Pan Afr Med J. (2014) 18 Suppl 1:3. doi: 10.11604/pamj.supp.2014.18.1.4608 PMC4199354 25328622

[5] Wandeler G Johnson LF Egger M . Trends in life expectancy of HIV-positive adults on antiretroviral therapy across the globe: comparisons with general population. Curr Opin HIV AIDS. (2016) 11:492–500. doi: 10.1097/COH.0000000000000298 27254748 PMC5055447

[6] Coghill AE Pfeiffer RM Shiels MS Engels EA . Excess mortality among HIV-infected individuals with cancer in the United States. Cancer Epidemiol Biomarkers Prev. (2017) 26:1027–33. doi: 10.1158/1055-9965.EPI-16-0964 28619832 PMC5500417

[7] Yarchoan R Uldrick TS . HIV-associated cancers and related diseases. N Engl J Med. (2018) 378:1029–41. doi: 10.1056/NEJMra1615896 29539283 PMC6890231

[8] Gopal S Achenbach CJ Yanik EL Dittmer DP Eron JJ Engels EA . Moving forward in HIV-associated cancer. J Clin Oncol. (2014) 32:876–80. doi: 10.1200/JCO.2013.53.1376 24550416 PMC3948093

[9] Shiels MS Engels EA . Evolving epidemiology of HIV-associated malignancies. Curr Opin HIV AIDS. (2017) 12:6–11. doi: 10.1097/coh.0000000000000327 27749369 PMC5240042

[10] Lv T Cao W Li T . HIV-related immune activation and inflammation: current understanding and strategies. J Immunol Res. (2021) 2021:7316456. doi: 10.1155/2021/7316456 34631899 PMC8494587

[11] Dwyer-Lindgren L Cork MA Sligar A Steuben KM Wilson KF Provost NR . Mapping HIV prevalence in sub-Saharan Africa between 2000 and 2017. Nature. (2019) 570:189–93. doi: 10.1038/s41586-019-1200-9 31092927 PMC6601349

[12] Ogoina D Onyemelukwe G Musa BO Babadoko A . Seroprevalence and determinants of human herpes virus 8 infection in adult Nigerians with and without HIV-1 infection. Afr Health Sci. (2011) 11:158–62. PMC3158522 21857844

[13] Jedy-Agba EE Oga EA Odutola M Abdullahi YM Popoola A Achara P . Developing national cancer registration in developing countries - case study of the Nigerian National System of Cancer Registries. Front Public Health. (2015) 3:186. doi: 10.3389/fpubh.2015.00186 26284233 PMC4519655

[14] National Agency for the Control of AIDS . National HIV and AIDS strategic framework 2021–2025 (2021). Available online at: https://naca.gov.ng/wp-content/uploads/2022/03/National-HIV-and-AIDS-Strategic-Framework-2021-2025-Final.pdf (Accessed August 4, 2026).

[15] Baethge C Goldbeck-Wood S Mertens S . SANRA—a scale for the quality assessment of narrative review articles. Res Integr Peer Rev. (2019) 4:5. doi: 10.1186/s41073-019-0064-8 30962953 PMC6434870

[16] National Agency for the Control of AIDS . Nigeria HIV/AIDS Indicator and Impact Survey (NAIIS) 2018: National summary sheet (preliminary findings) (2019). Available online at: https://naca.gov.ng/wp-content/uploads/2021/02/NAIIS-PA-NATIONAL-FACTSHEET-FINAL.pdf (Accessed August 4, 2026).

[17] Jedy-Agba EE Curado MP Oga E Samaila MO Ezeome ER Obiorah C . The role of hospital-based cancer registries in low and middle income countries-the Nigerian case study. Cancer Epidemiol. (2012) 36:430–5. doi: 10.1016/j.canep.2012.05.010 22704971 PMC3438360

[18] Edington G Maclean C . A cancer rate survey in Ibadan, Western Nigeria, 1960-63. Br J Cancer. (1965) 19:471–81. doi: 10.1038/bjc.1965.55 5833601 PMC2071349

[19] Silas OA Musa J Afolaranmi TO Sagay AS Evans CT Achenbach CJ . Predictors of mortality from a population-based cancer registry data in Jos, Nigeria: a resource-limited setting. Front Med (Lausanne). (2020) 7:227. doi: 10.3389/fmed.2020.00227 32582731 PMC7287203

[20] Zubairu IH Balogun MS . Population-based cancer registries in Nigeria and the National Cancer Control Programme. Ecancermedicalscience. (2023) 17:1592. doi: 10.3332/ecancer.2023.1592 37799955 PMC10550292

[21] Jedy-Agba E Curado MP Ogunbiyi O Oga E Fabowale T Igbinoba F . Cancer incidence in Nigeria: a report from population-based cancer registries. Cancer Epidemiol. (2012) 36:e271–8. doi: 10.1016/j.canep.2012.04.007 22621842 PMC3438369

[22] Mohammed AZ Edino ST Ochicha O Gwarzo AK Samaila AA . Cancer in Nigeria: a 10-year analysis of the Kano cancer registry. Niger J Med. (2008) 17:280–4. doi: 10.4314/njm.v17i3.37396 18788253

[23] Akarolo-Anthony SN Maso LD Igbinoba F Mbulaiteye SM Adebamowo CA . Cancer burden among HIV-positive persons in Nigeria: preliminary findings from the Nigerian AIDS-cancer match study. Infect Agent Cancer. (2014) 9:1. doi: 10.1186/1750-9378-9-1 24597902 PMC3942812

[24] Federal Ministry of Health, Nigeria . National Cancer Control Plan 2018–2022. Abuja: Federal Ministry of Health (2018). Available online at: https://www.iccp-portal.org/sites/default/files/plans/NCCP_Final%20%5B1%5D.pdf (Accessed August 4, 2026).

[25] Ocheni S Aken'Ova YA . Association between HIV/AIDS and malignancies in a Nigerian tertiary institution. West Afr J Med. (2004) 23:151–5. doi: 10.4314/wajm.v23i2.28109 15287296

[26] Musa J Kocherginsky M Magaji FA Maryam AJ Asufi J Nenrot D . Epidemiology and survival outcomes of HIV-associated cervical cancer in Nigeria. Infect Agents Cancer. (2023) 18:68. doi: 10.1186/s13027-023-00550-7 37915091 PMC10619301

[27] Daniel GO Musa J Akindigh TM Shinku F Shuaibu SI Kwaghe B . Prevalence and predictors of precancerous cervical lesions among HIV-positive women in Jos, north-central Nigeria. Int J Gynaecol Obstet. (2020) 151:253–9. doi: 10.1002/ijgo.13312 32683675

[28] Anorlu RI Igwilo CI Akanmu AS Banjo AA Odunukwe NN Okany CC . Prevalence of abnormal cervical smears among patients with HIV in Lagos, Nigeria. West Afr J Med. (2007) 26:143–7. 17939318

[29] Awolude OA Oyerinde SO . Invasive cervical cancer in Ibadan: socio-sexual characteristics, clinical stage at presentation, histopathology distributions and HIV status. Afr J Infect Dis. (2018) 13:32–8. doi: 10.21010/ajid.v13i1.4 30596194 PMC6305076

[30] Nyam CJ Musa J Joyce BT Kim K Wang J Green SJ . Molecular epidemiology of human papillomavirus genotypes among HIV-negative women and women living with HIV with cervical cancer in two regions in Nigeria. Int J STD AIDS. (2025) 36:1018–27. doi: 10.1177/09564624251362592 40820278

[31] Zheng Y Han J Qu Y Wang J Joyce BT Okeke E . Circulating cell-free DNA methylation biomarkers for hepatocellular carcinoma risk prediction in HIV-positive Nigerian population. Int J Cancer. (2026) 158:1281–91. doi: 10.1002/ijc.70204 41129333

[32] Okeke E Mark Davwar P Mullen B Duguru M Agbaji O Sagay A . The impact of HIV on hepatocellular cancer survival in Nigeria. Trop Med Int Health. (2021) 26:335–42. doi: 10.1111/tmi.13532 33244817

[33] Davwar PM Okeke E Duguru M Nyam D Bell K Odeghe EA . Hepatocellular carcinoma presentation and prognosis among Nigerian adults with and without HIV. PloS One. (2023) 18:e0282539. doi: 10.1371/journal.pone.0282539 36877687 PMC9987777

[34] Adebamowo CA Ajayi OO . Breast cancer in Nigeria. West Afr J Med. (2000) 19:179–91. 11126081

[35] Otegbayo JA Taiwo BO Akingbola TS Odaibo GN Adedapo KS Penugonda S . Prevalence of hepatitis B and C seropositivity in a Nigerian cohort of HIV-infected patients. Ann Hepatol. (2008) 7:152–6. doi: 10.1016/s1665-2681(19)31872-1 18626434

[36] Grover S Martei YM Puri P Prabhakar P Mutebi M Balogun OD . Breast cancer and HIV in Sub-Saharan Africa: a complex relationship. J Glob Oncol. (2018) 4:1–11. doi: 10.1200/JGO.2016.006585 30241185 PMC6180795

[37] Salawu L Bolarinwa RA Lawal OO Oyekunle AA Adeodu O Adejuyigbe EA . AIDS-related lymphomas in Nigeria an emerging phenomenon. Infect Agent Cancer. (2010) 5:A3. doi: 10.1186/1750-9378-5-S1-A338164791

[38] Oluwasola AO Olaniyi JA Otegbayo JA Ogun GO Akingbola TS Ukah CO . A fifteen-year review of lymphomas in a Nigerian tertiary healthcare centre. J Health Popul Nutr. (2011) 29:310–6. doi: 10.3329/jhpn.v29i4.8446 21957669 PMC3190361

[39] Bolarinwa RA Ndakotsu MA Oyekunle AA Salawu L Akinola NO Durosinmi MA . AIDS-related lymphomas in Nigeria. Braz J Infect Dis. (2009) 13:359–61. doi: 10.1590/S1413-86702009000500009 20428636

[40] Ibanga A Ekanem IA Nkanga DG Asana UE Duke RE Etim BA . Human immunodeficiency virus (HIV) related conjunctival tumors. Int J Ophthalmic Pathol. (2017) 6:4. doi: 10.4172/2324-8599.1000209

[41] Fasina O . Ocular surface squamous neoplasia at a tertiary eye facility, Southwestern Nigeria: a 10-year review. Int Ophthalmol. (2021) 41:3325–31. doi: 10.1007/s10792-021-01894-y 34014459

[42] Akanbi MO Bilaver LA Achenbach C Hirschhorn LR Rivera AS Silas OA . Analyses of Kaposi sarcoma trends among adults establishing initial outpatient HIV care in Nigeria: 2006–2017. Infect Agents Cancer. (2022) 17:10. doi: 10.1186/s13027-022-00424-4 35313941 PMC8935748

[43] Akanbi MO Bilaver LA Achenbach C Hirschhorn LR Rivera AS Adekolujo OS . Incident Kaposi sarcoma during the expansion of antiretroviral therapy eligibility in Nigeria: a retrospective cohort study. BMC Cancer. (2023) 23:890. doi: 10.1186/s12885-023-11402-3 37735371 PMC10512500

[44] Mandong B Chirdan L Anyebe A Mannaseh A . Histopathology of Kaposi’s sarcoma in Jos: a 16-year review. Ann Afr Med. 3:174–6. Available online at: https://www.ajol.info/index.php/aam/article/view/8333.

[45] Agaba PA Sule HM Ojoh RO Hassan Z Apena L Mu'azu MA . Presentation and survival of patients with AIDS-related Kaposi's sarcoma in Jos, Nigeria. Int J STD AIDS. (2009) 20:410–3. doi: 10.1258/ijsa.2008.008353 19451327

[46] Akinde O Obadofin O Adeyemo T Omoseebi O Ikeri N Okonkwo I . Kaposi sarcoma among HIV infected patients in Lagos University Teaching Hospital, Nigeria: a 14-year retrospective clinicopathological study. J Skin Cancer. (2016), 9368023. doi: 10.1155/2016/9368023 27034839 PMC4808559

[47] Forae GD Obaseki DE . Trends and histopathological patterns of Kaposi sarcoma at the University of Benin Teaching Hospital, Benin City, Nigeria. Oman Med J. (2018) 33:337–41. doi: 10.5001/omj.2018.61 30038734 PMC6047182

[48] Asuquo ME Ogunkeyede A Bassey EE Ebughe G . Kaposi sarcoma: changing trend in Calabar, south eastern Nigeria. Ann Afr Med. (2008) 7:98–101. doi: 10.4103/1596-3519.55670 19253517

[49] Kagu MB Nggada HA Garandawa HI Askira BH Durosinmi MA . AIDS-associated Kaposi's sarcoma in Northeastern Nigeria. Singapore Med J. (2006) 47:1069–74. 17139404

[50] Silas OA Manasseh AN Musa J Dauda AM Zoakah AI Mandong BM . Changing patterns in trend of top 10 cancers in the Jos University Teaching Hospital's (JUTH) cancer registry (1996-2018), Jos, north-central Nigeria. J Med Trop. (2019) 21:81–6. doi: 10.4103/jomt.jomt_20_19 36467537 PMC9718006

[51] Freeman EE Semeere A McMahon DE Byakwaga H Laker-Oketta M Regan S . Beyond T staging in the "Treat-All" era: Severity and heterogeneity of Kaposi sarcoma in East Africa. J Acquir Immune Defic Syndr. (2021) 87:1119–27. doi: 10.1097/QAI.0000000000002699 33871409 PMC8263487

[52] Poizot-Martin I Brégigeon S Palich R Marcelin AG Valantin MA Solas C . Immune reconstitution inflammatory syndrome associated Kaposi sarcoma. Cancers (Basel). (2022) 14:986. doi: 10.3390/cancers14040986 35205734 PMC8869819

[53] Ahmed A Bugaje MA Babadoko AA Ameh E . Management of AIDS-associated Kaposi's sarcoma in Nigerian children: a case series and review of literature. J Natl Med Assoc. (2012) 104:385–9. doi: 10.1016/s0027-9684(15)30181-4 23092054

[54] Krown SE . Treatment strategies for Kaposi sarcoma in sub-Saharan Africa: challenges and opportunities. Curr Opin Oncol. (2011) 23:463–8. doi: 10.1097/CCO.0b013e328349428d 21681092 PMC3465839

[55] Shao D Wu P Jiang H Wang Z . Global trends and future projections of cervical cancer burden: an integrated analysis of GBD 2021, UN population and WHO HPV vaccination data. Front Public Health. (2026) 14:1702186. doi: 10.3389/fpubh.2026.1702186 41668843 PMC12883747

[56] Sowunmi A Alabi A Fatiregun O Olatunji T Okoro US Durosinmi Etti AF . Trend of cancer incidence in an oncology center in Nigeria. West Afr J Radiol. (2018) 25:52–6. doi: 10.4103/wajr.wajr_26_1742781439

[57] Stelzle D Tanaka LF Lee KK Ibrahim Khalil A Baussano I Shah ASV . Estimates of the global burden of cervical cancer associated with HIV. Lancet Glob Health. (2021) 9:e161–9. doi: 10.1016/S2214-109X(20)30459-9 33212031 PMC7815633

[58] Ezechi OC Ostergren PO Nwaokorie FO Ujah IA Odberg Pettersson K . The burden, distribution and risk factors for cervical oncogenic human papilloma virus infection in HIV positive Nigerian women. Virol J. (2014) 11:5. doi: 10.1186/1743-422X-11-5 24433568 PMC3896716

[59] Kabuga AI Nejati A Arero AG Jalilvand S Mokhtari-Azad T Sighaldeh SS . Prevalence and type distribution of human papillomavirus recovered from the uterine cervix of Nigerian women: a systematic review and meta-analysis. Asian Pac J Cancer Prev. (2020) 21:2837–46. doi: 10.31557/APJCP.2020.21.10.2837 33112538 PMC7798145

[60] Akarolo-Anthony SN Al-Mujtaba M Famooto AO Dareng EO Olaniyan OB Offiong R . HIV associated high-risk HPV infection among Nigerian women. BMC Infect Dis. (2013) 13:521. doi: 10.1186/1471-2334-13-521 24192311 PMC3826514

[61] Musa J Achenbach C Taiwo B Berzins B Silas O Daru PH . High-risk human papilloma virus and cervical abnormalities in HIV-infected women with normal cervical cytology. Infect Agent Cancer. (2014) 9:36. doi: 10.1186/1750-9378-9-36 25395987 PMC4230523

[62] Abdus-Salam AA Yusuf SA Ehiedu ECG Adeleke OK . Human immunodeficiency virus in cervical cancer patients treated with radiotherapy in Ibadan, Nigeria: A revisit. Ann Ib Postgrad Med. (2025) 23:83–7. PMC12704350 41404081

[63] Atibioke OP Oyasope BT Ojomo OA Jima BW Akewusola K Ogunkayode J . A cross-sectional assessment of cervical cancer vaccination and screening among women of reproductive age living with HIV in North Central States, Nigeria. Pan Afr Med J. (2025) 52:175. doi: 10.11604/pamj.2025.52.175.49492 41939522 PMC13044238

[64] Habila MA Sagay E Obeng-Kusi M Ali MJ Magaji FA Shambe IH . Utilization of opportunistic cervical cancer screening in Nigeria. Cancer Causes Control. (2024) 35:9–20. doi: 10.1007/s10552-023-01764-1 37530986

[65] Chigbu CO Dim CC Eneh SC Olakunde BO Olawepo JO Onyeka TC . Cervical cancer screen-and-treat services at HIV treatment sites in Nigeria: findings from the ICON-3 Practice Based Research Network. BMC Health Serv Res. (2026) 26:412. doi: 10.1186/s12913-026-14167-0 41715108 PMC13032365

[66] Ezechi OC Gab-Okafor CV Ostergren PO Odberg Pettersson K . Willingness and acceptability of cervical cancer screening among HIV positive Nigerian women. BMC Public Health. (2013) 13:46. doi: 10.1186/1471-2458-13-46 23327453 PMC3567931

[67] World Health Organization . WHO Guidelines for the Use of Thermal Ablation for Cervical Pre-Cancer Lesions. Geneva: World Health Organization (2019). Available online at: https://www.ncbi.nlm.nih.gov/books/NBK549179/ (Accessed August 4, 2026). 31661202

[68] Federal Ministry of Health, Nigeria . National Strategic Plan for the Prevention and Control of Cancer of the Cervix in Nigeria (2023–2027). Abuja, Nigeria: Federal Ministry of Health (2023). Available online at: https://www.iccp-portal.org/resources/national-strategic-plan-prevention-and-control-cancer-cervix-nigeria (Accessed August 4, 2026).

[69] Silas OA Achenbach CJ Hou L Murphy RL Egesie JO Sagay SA . Outcome of HIV-associated lymphoma in a resource-limited setting of Jos, Nigeria. Infect Agent Cancer. (2017) 12:34. doi: 10.1186/s13027-017-0144-7 28592989 PMC5460353

[70] Shiels MS Cole SR Kirk GD Poole C . A meta-analysis of the incidence of non-AIDS cancers in HIV-infected individuals. J Acquir Immune Defic Syndr. (2009) 52:611–22. doi: 10.1097/QAI.0b013e3181b327ca 19770804 PMC2790038

[71] Yuan T Hu Y Zhou X Yang L Wang H Li L . Incidence and mortality of non-AIDS-defining cancers among people living with HIV: A systematic review and meta-analysis. EClinicalMedicine. (2022) 52:101613. doi: 10.1016/j.eclinm.2022.101613 35990580 PMC9386399

[72] Machalek DA Poynten M Jin F Fairley CK Farnsworth A Garland SM . Anal human papillomavirus infection and associated neoplastic lesions in men who have sex with men: a systematic review and meta-analysis. Lancet Oncol. (2012) 13:487–500. doi: 10.1016/S1470-2045(12)70080-3 22445259

[73] Volpi CR Chama J Mansfield ME Adebiyi R Mitchell A Aigoro JA . Strengthening anal cancer prevention in Abuja, Nigeria: Identifying barriers and potential strategies to improve training. PloS Global Public Health. (2025) 5:e0004616. doi: 10.1371/journal.pgph.0004616 40601571 PMC12221040

[74] Abraham AG D'Souza G Jing Y Gange SJ Sterling TR Silverberg MJ . Invasive cervical cancer risk among HIV-infected women: a North American multicohort collaboration prospective study. J Acquir Immune Defic Syndr. (2013) 62:405–13. doi: 10.1097/QAI.0b013e31828177d7 23254153 PMC3633634

[75] Purohit S Zhi W Ferris DG Alverez M Tran LKH Tran PMH . Senescence-associated secretory phenotype determines survival and therapeutic response in cervical cancer. Cancers (Basel). (2020) 12:2899. doi: 10.3390/cancers12102899 33050319 PMC7601905

[76] Okoye JO Ofodile CA Adeleke OK Obioma O . Prevalence of high-risk HPV genotypes in sub-Saharan Africa according to HIV status: a 20-year systematic review. Epidemiol Health. (2021) 43:e2021039. doi: 10.4178/epih.e2021039 34044477 PMC8510839

[77] Ogunsola OO Ajayi OA Ojo TO Osayi E Wudiri K Amoo B . Cervical cancer screening and treatment for PLWHIV: experiences from an innovative program in Nigeria. Reprod Health. (2023) 20:125. doi: 10.1186/s12978-023-01658-0 37633884 PMC10463408

[78] Akinwande AM Ugwuanyi DC Chiegwu HU Idigo F Ogolodom MP Anakwenze CP . Radiotherapy services in low resource settings: The situation in Nigeria. SAGE Open Med. (2023) 11:1–8. doi: 10.1177/20503121231153758 36778199 PMC9909043

[79] U.S. President’s Emergency Plan for AIDS Relief (PEPFAR) . Nigeria country operational plan (COP 2023) strategic direction summary (2023). Available online at: https://www.prepwatch.org/wp-content/uploads/2024/06/Nigeria.pdf (Accessed August 4, 2026).

